# Population synchrony indicates functional connectivity in a threatened sedentary butterfly

**DOI:** 10.1007/s00442-023-05357-2

**Published:** 2023-03-28

**Authors:** Alex Blomfield, Rosa Menéndez, Andrew Wilby

**Affiliations:** Lancaster Environment Centre, Lancaster University, Lancaster, LA1 4YQ UK

**Keywords:** Dispersal, Ecological succession, Habitat permeability, Insect conservation, *Boloria euphrosyne*

## Abstract

Dispersal is a key influence on species’ persistence, particularly in the context of habitat fragmentation and environmental change. Previously, residual population synchrony has been demonstrated to be an effective proxy for dispersal in mobile butterflies (Powney et al. 2012). Here, we highlight the utility and limitations of population synchrony as an indicator of functional connectivity and persistence, at a range of spatial scales, in a specialist, sedentary butterfly. While at the local scale, population synchrony is likely indicative of dispersal in the pearl-bordered fritillary, *Boloria euphrosyne,* over larger scales, habitat is likely to influence population dynamics. Although declines in local-scale synchrony conformed to typical movement in this species, synchrony showed no significant trend with distance when studied at larger (between-site) scales. By focusing on specific site comparisons, we draw the conclusion that heterogeneity in habitat successional stage drives asynchrony between sites at larger distances and is, therefore, likely to be a more important driver of population dynamics over large distances than dispersal. Within-site assessments of synchrony highlight differences in dispersal based on habitat type, with movement shown to be most inhibited between transect sections with contrasting habitat permeability. While synchrony has implications for metapopulation stability and extinction risk, no significant difference was found in average site synchrony between sites that had gone extinct during the study period and those remaining occupied. We demonstrate that population synchrony may be used to assess local-scale movement between sedentary populations, as well as to understand barriers to dispersal and guide conservation management.

**Supplementary Information:**

The online version contains supplementary material available at 10.1007/s00442-023-05357-2.

## Introduction

Dispersal is critical for population persistence in fragmented habitats (Hanski 1999). Understanding how landscape connectivity facilitates dispersal is crucial for predicting how species will respond to habitat fragmentation and environmental change, as well as for informing the design and management of reserves (Fourcade and Öckinger 2017). In the face of climate change, landscape connectivity can promote persistence by facilitating range expansions, while local-scale movement between heterogeneous habitat patches allows species to select microclimates that can buffer against extreme events (Saura et al. 2014; Suggitt et al. 2018). Movement of individuals between sub-populations is also key for metapopulation stability as it enables the rescue of dwindling populations and recolonisation of empty habitat patches (Hanski 1998).

Given the significance of dispersal, it is important to be able to assess functional connectivity (the extent to which habitat structure facilitates or impedes movement of a species through the landscape) (Tischendorf and Fahrig 2000; Hanski et al. 2004). While landscape structure may be used to infer dispersal, it describes habitat connectivity irrespective of species’ use (Powney et al. 2012). Indeed, physical landscape features, such as corridors, do not necessarily reflect realised dispersal (Tischendorf and Fahrig 2000). Given the limitations of structural connectivity for making predictions about realised dispersal, it is often necessary to study species’ movements to understand functional connectivity. Butterflies provide good model organisms for studying dispersal and environmental change (Stephens et al. 2010). However, the conventional methods for assessing dispersal, such as mark–release–recapture studies, are time-consuming and labour intensive, meaning that their spatial coverage is often limited. Such studies may also overlook long-distance dispersal events, which, though rare, can have a profound impact on population persistence and genetic structure (Zimmermann et al. 2011). An alternative method to describe functional connectivity is population synchrony, the correlation in annual variation in abundance between separate populations. As population dynamics are more closely synchronised in populations connected by frequent movement of individuals, synchrony is an indicator of dispersal (Oliver et al. 2017). Indeed, population synchrony has been shown to correlate with factors affecting dispersal, such as landscape permeability and prevailing weather conditions and to decline with distance between sites (Thomas 1991; Powney et al. 2012; Vindstad et al. 2019). For example, in the silver-studded blue, *Plebejus argus*, synchrony between local patches declined with distance, breaking down beyond 600 m (Thomas 1991). However, in addition to dispersal, several other factors can synchronise population dynamics. Synchrony has been shown to be higher in populations at the edges of species range margins, as well as being driven by correlation in environmental conditions at geographically close sites (the Moran effect) and trophic interactions (Royama 1992; Powney et al. 2010; Kahilainen et al. 2018). Natural enemies can have a profound impact on host abundance; for example, the parasitic wasp, *Apanteles bignellii,* is thought to drive strong inter-annual population fluctuations in the marsh fritillary, *Euphydryas aurinia* (Porter 1983).

The role of microclimate similarities and trophic interactions in driving synchrony is difficult to account for. However, by controlling for the synchronising effects of large-scale, shared climatic conditions on populations, residual synchrony has been used as an effective proxy for butterfly dispersal at a range of spatial scales (Powney et al. 2011, 2012). Furthermore, by comparing population synchrony in different landscape contexts, landscape features that promote or pose barriers to dispersal can be determined. For example, distance along forest edge has been found to be a better predictor of synchrony between ringlet, *Aphantopus hyperantus,* populations than direct distance between patches (Powney et al. 2012). In the speckled wood, *Pararge aegeria,* residual synchrony was also shown to be greater between patches where the intervening matrix was more permeable to dispersal; however, matrix suitability was of greatest importance for dispersal between sites over 20 km apart (Powney et al. 2011). Unlike geographically close sites, which are likely to be readily colonisable by this mobile butterfly, regardless of matrix characteristics, further apart sites are likely to be more reliant on matrix features to facilitate successful dispersal.

It has previously been suggested that given the limited dispersal propensity of many butterfly species, dispersal will be insufficient to synchronise their dynamics at large spatial scales, although there is evidence that habitat stepping stones can synchronise population dynamics over long distances (Pollard and Yates 1993; Powney et al. 2011). For example, in mobile species such as *P. aegeria,* dispersal is thought to drive synchrony between populations up to 160 km apart (Powney et al. 2011). However, synchrony has been shown to decline more rapidly with distance in sedentary populations than in those that are more mobile (Sutcliffe et al. 1996). Nevertheless, population synchrony has also been demonstrated to be indicative of dispersal operating at smaller scales, such as within sites (Powney et al. 2012). This has also been demonstrated empirically with mark–release–recapture data. For example, in the bog fritillary, *Boloria eunomia*, synchrony was correlated with the frequency of inter-patch movements (Oliver et al. 2017). The presence of distance decay in synchrony at these small spatial scales suggests that population synchrony may be relevant for understanding dispersal in sedentary butterflies, in addition to more mobile species. While the previous studies linking residual synchrony to dispersal have often focused on mobile, generalist butterflies, a better understanding of within-site movement would aid conservation management for sedentary butterflies, which are overrepresented in declining species (Powney et al. 2012; Eskildsen et al. 2015; Warren et al. 2021). In addition, population synchrony may be used to highlight the impacts of environmental change on populations. Previously, encroaching forest has been associated with the decoupling of population dynamics, due to reduced dispersal between populations of the Rocky Mountain Apollo butterfly, *Parnassius smintheus* (Roland and Matter 2007). Population synchrony also has important implications for extinction risk. Local extinction events can drive synchrony, resulting in positive feedback in extinction risk. This was demonstrated by the experimental removal of *P. smintheus* from two sub-populations, which resulted in a marked increase in synchrony between remaining populations in the network (Matter and Roland 2010). Local extinction results in a decline in immigration across the network. Where this leads to a simultaneous reduction in abundance in remaining populations, it can synchronise their dynamics (Matter and Roland 2010). Asynchrony at the metapopulation level promotes persistence as it can buffer species against regional extinction in suboptimal years and surviving populations can act as sources for recolonisation (Hanski 1998; Kahilainen et al. 2018). Even though dispersal promotes local-scale persistence, it also contributes to the synchronising of population dynamics. In this way, population synchrony is understood to pose something of a ‘double edged sword’ to extinction risk (Hudson and Cattadori 1999). As there is likely to be an optimum level of synchrony for long-term metapopulation persistence, a greater understanding of the links between population synchrony and extinction risk could help to predict population vulnerability to extinction, allowing management to be targeted accordingly (Powney et al. 2010).

Here, we investigate the use of population synchrony as an indicator of functional connectivity in a sedentary, specialist butterfly, the pearl-bordered fritillary, *Boloria euphrosyne.* Residual synchrony (synchrony after controlling for climatic factors) was assessed in the Morecambe Bay region, in north-west England, at two spatial scales, within- and between-sites. Within the maximum dispersal distance of the study species (< 4.5 km), it was predicted that population synchrony would be indicative of dispersal and therefore would decline with distance. At larger scales (> 4.5 km), the frequency of dispersal was hypothesised to be insufficient to synchronise population dynamics, and instead, habitat similarity was predicted to synchronise dynamics. While *B. euphrosyne* is described as sedentary butterfly, landscape structure is reported to influence mobility, with open habitat likely to facilitate dispersal (Barnett and Warren 1995). To investigate this, we used population synchrony to assess the influence of landscape permeability on dispersal in this species. We predicted that synchrony would be dependent on habitat structure, such that it would display a stronger distance-decay effect in habitats with a closed structure than in those with an open habitat structure. Additionally, we investigated the role of site-specific factors in driving population dynamics. We hypothesised that extrinsic factors, such as vegetation successional stage, may drive asynchrony between management units. Finally, the implications of synchrony for extinction risk were investigated. As inter-patch movements are likely to be infrequent, yet important for population persistence, it was hypothesised that populations from sites with higher average synchrony would have a lower risk of extinction.

## Materials and methods

**Fig. 1:**
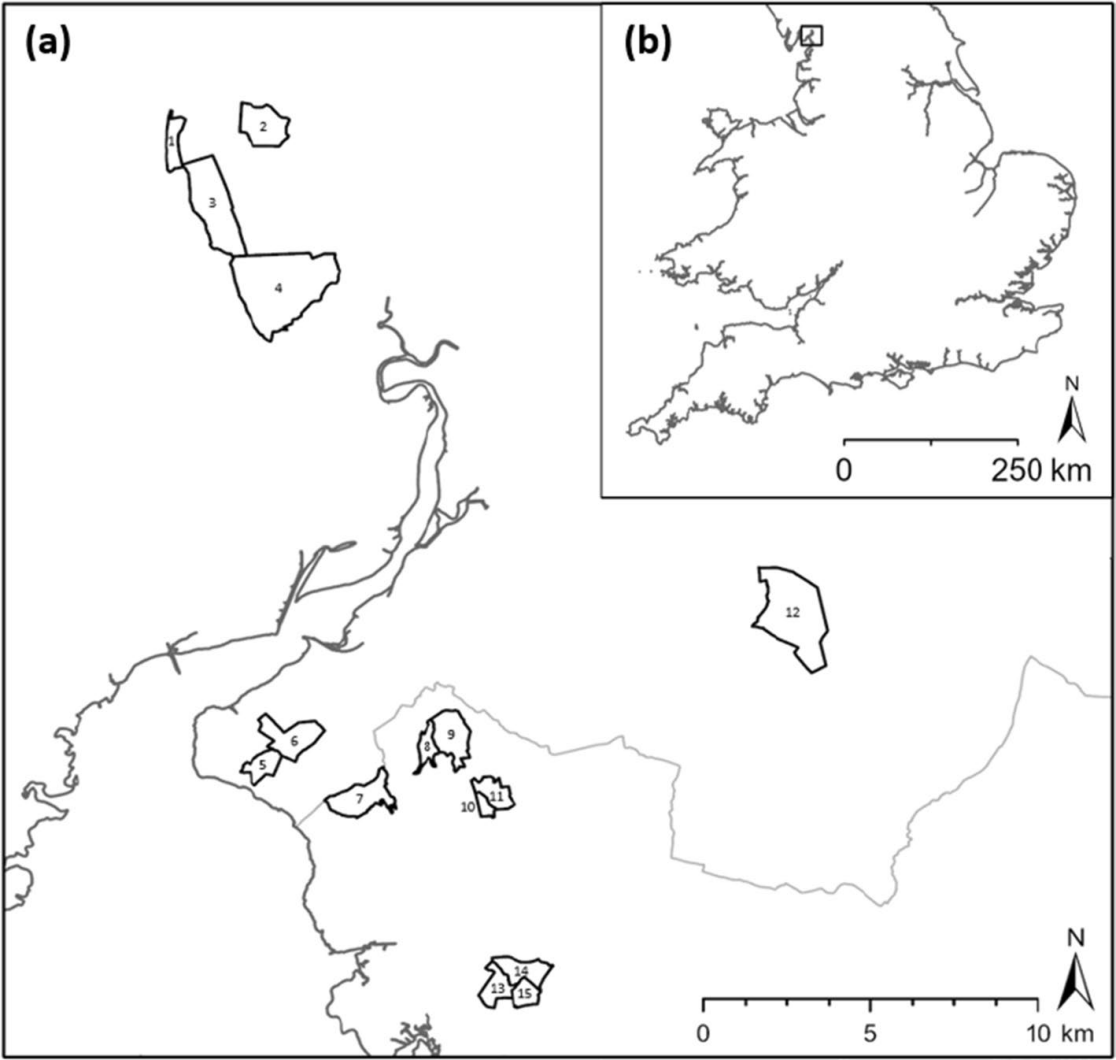
Location of study sites in **a** local and **b** national context. County boundaries (Lancashire and Cumbria) are shown in grey. See Online Resource 1 for transect site details

Butterfly abundance data were obtained from the United Kingdom Butterfly Monitoring Scheme (UKBMS), which is a long-standing programme of coordinated monitoring under which butterflies are recorded along fixed route transects (Pollard and Yates 1993). Residual population synchrony, over the 1978–2016 period, was assessed in *B. euphrosyne* populations at 15 UKBMS transect sites in the Morecambe Bay region (Fig. 1; Online Resource 1). Transect route maps for each site were provided by Butterfly Conservation. Butterfly transects are split into sections usually based on broad habitat type or landscape features (for the study sites, these ranged from 7 to 15 sections per transect, with an average length of approximately 300 m). Analysis was performed at both the site and transect-section level. Only site or section pairs that had at least 7 years of common data were included in analysis (Powney et al. 2011). As a chain of zero counts over the time series can inflate synchrony values, years with zero counts that were not bounded by a positive value were also excluded (Powney et al. 2011). Site-level analysis included pairwise comparisons across 15 sites. Section-level synchrony was assessed for 106 transect sections from 13 sites (sites 1 and 6 were excluded from section-level analysis due to transect sections at these sites having fewer than 7 years of data where zero counts were bounded by a positive value).

Butterfly transects are recorded weekly from April 1st until September, but where minimum weather criteria are not met, missing data may be present. UKBMS site indices are calculated using a general additive model, to account for missing data (Botham et al. 2020). To account for differential recording effort between transects when analysing synchrony at the section level, section abundance indices calculated proportionally from the UKBMS index for the whole site were used. Weekly counts from each transect section were summed to calculate the proportional contribution of each section to the site’s annual abundance. Where multiple records for a site were submitted in a single week, counts were averaged. UKBMS site indices were multiplied by the section proportional contribution, to produce section-level indices. Where recording effort was insufficient for the UKBMS index to have been calculated, those comparisons were excluded.

Correlation of long-term population trends in a region could inflate synchrony (Paradis et al. 1999). To control for such trends, residuals from a linear model of index and year were extracted and used in both site- and section-level analysis (Powney et al. 2011). The data were also ‘Pre-whitened’ to account for the role of climate in synchronising population dynamics (Powney et al. 2012). To achieve this, the following equation was used:

$$d_{it} \; = \;c_{it} \; - \;m_{i} I_{t} ,$$

*d*_*it*_ is the pre-whitened count for site or transect section *i* in year *t*, *c*_*it*_ is the raw abundance count for *i* in year *t*, *m*_*i*_ is the mean abundance over the study period, and *I*_*t*_ is the national population index in year *t*. The national indices used were the English UKBMS national log collated indices for *B. euphrosyne* (Botham et al. 2019). These national indices were standardised using the following equation:

$$I_{t} \; = \;\left( {n_{t} \; - \;m} \right)\; + \;1,$$

where *m*, the overall mean of national index values for the 1978–2016 period, was subtracted from each annual value, *n*. Population synchrony was calculated using the pre-whitened, standardised abundance data as the Spearman’s rank correlation coefficient for transect site and section pairs. The study sites were classified by population status and dominant habitat type. Population status was classed as extinct if *B. euphrosyne* was absent from the transect for three consecutive years, up to and including 2018. Habitat type was defined at the site and transect-section level as the most frequently occurring habitat type, using the 2015 Land Cover Map (Rowland et al. 2017; Online Resource 1). For the analysis, habitat similarity was classified using these land-cover categories, with “1” referring to paired transect sites or sections sharing the same broad habitat type and “0” to those with different habitat classifications. Also based on the classifications of broad habitat type, transect sections were classed as having either an “open” or “closed” habitat structure. Sections with a dominant habitat type of grassland or arable have a high permeability to dispersal and were classed as “open,” while woodland habitats were classed as “closed”. As the analysis involved pairwise comparisons between transect sections, this classification resulted in three categories of habitat structure, with “open” or “closed” describing pairwise comparisons between sections that shared the same habitat structure, while “mixed” described transect section pairs with contrasting habitat structures (“open” vs “closed”). Distance between transect pairs was calculated in ArcGIS Pro 2.5.0 as the Euclidean distance between site and transect section midpoints (Esri 2020).

### Statistical analysis

For the site-level analysis, linear models were used to examine the relationship between population synchrony and distance. Dispersal has been reported to synchronise population dynamics over large spatial scales (Powney et al. 2011). However, *B. euphrosyne* is a sedentary species and although it has been reported to move up to 4.5 km between sites, such long-distance dispersal events are rare (Barnett and Warren 1995). For this reason, separate models were built to assess synchrony between all pairwise site comparisons and for those less than 4.5 km apart. Habitat similarity was included as an explanatory variable.

Average synchrony was calculated at the site level and related to population status (occupied or extinct) using a generalised linear model with binomial errors and a logit link function.

Population synchrony was also investigated at the transect-section level. A generalised additive model (GAM) was fitted to investigate threshold effects in the relationship between synchrony and distance between transect sections less than 4.5 km apart.

Synchrony between transect sections was also examined at key sites (sites 2, 8, 9, 14, and 15; Online Resource 1) to investigate drivers of asynchrony between sites at longer distances. Key sites were those which had over 350 data points. Here, linear mixed-effects models were built to incorporate all pairwise comparisons for the selected site. The site of each corresponding transect section was included as a random effect.

Linear mixed-effects models were also used to assess the influence of habitat and distance on local synchrony, by assessing synchrony within-sites, at the transect section level. Transect site was included as a random effect. In addition to building a model for all within-site pairwise comparisons (*n* = 515), the effect of habitat structure was investigated. The data were split into section pairs where both sections were “open” (n = 50), both were “closed” (n = 388) or “mixed” (n = 77), where one section was closed and the other open, with separate models built for each category. All analysis was carried out in R Studio Version 4.0.3 (R Core Team 2020).

## Results

### Population synchrony

**Fig. 2:**
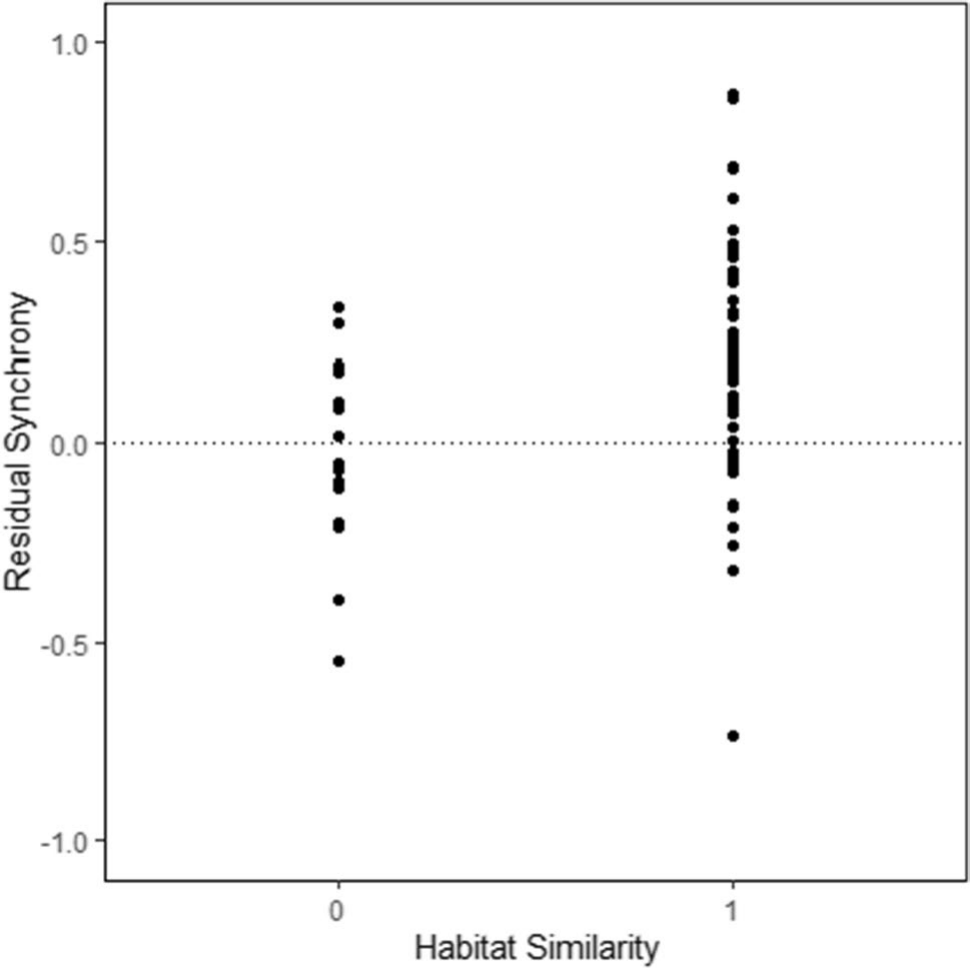
Residual population synchrony and habitat similarity for all transect site pairs. Habitat similarity is classed as “1” for site pairs which share the same category of broad habitat type and as “0” for sites of different habitat types. Above the dashed line, population dynamics are synchronised; below the line, dynamics are asynchronous

The relationship between population synchrony and distance was non-significant for pairwise comparisons at the site level. However, habitat similarity was shown to have a significant effect on population synchrony over these large spatial scales, with greater synchrony between sites sharing the same habitat type (Table 1a; Fig. 2). As the reported maximum dispersal distance for *B. euphrosyne* is 4.5 km, population synchrony was also assessed for transect sites within this distance. There was some evidence that synchrony of populations located less than 4.5 km apart may decline with distance, though this was only marginally significant (Table 1b; Online Resource 2). Site-level synchrony was also not significantly influenced by habitat similarity at this scale (Table 1b). Population synchrony was not related to extinction risk, with no significant difference found in average synchrony between populations classed as “extinct” or “occupied” (estimate = 1.71; std. error = 3.31; *z* = 0.52; *p* value = 0.61; *d.f* = 13).

**Table 1:** Significance of distance and habitat on between-site population synchrony for (a) all sites and (b) sites less than 4.5 km apart. Habitat similarity is classed as “1” for site pairs which share the same category of broad habitat type and as “0” for sites of different habitat types

Predictor	Coefficient	SE	*t*	*p*
a) All sites				
Distance (km)	0.006	0.007	0.859	0.393
Habitat similarity		0.078	3.110	**0.003**
0	− 0.071			
1	0.172			
b) < 4.5 km Apart				
Distance (km)	− 0.089	0.046	− 1.920	0.064
Habitat similarity		0.238	0.560	0.579
0	0.248			
1	0.382			

Significant relationships are highlighted in bold

**Fig. 3:**
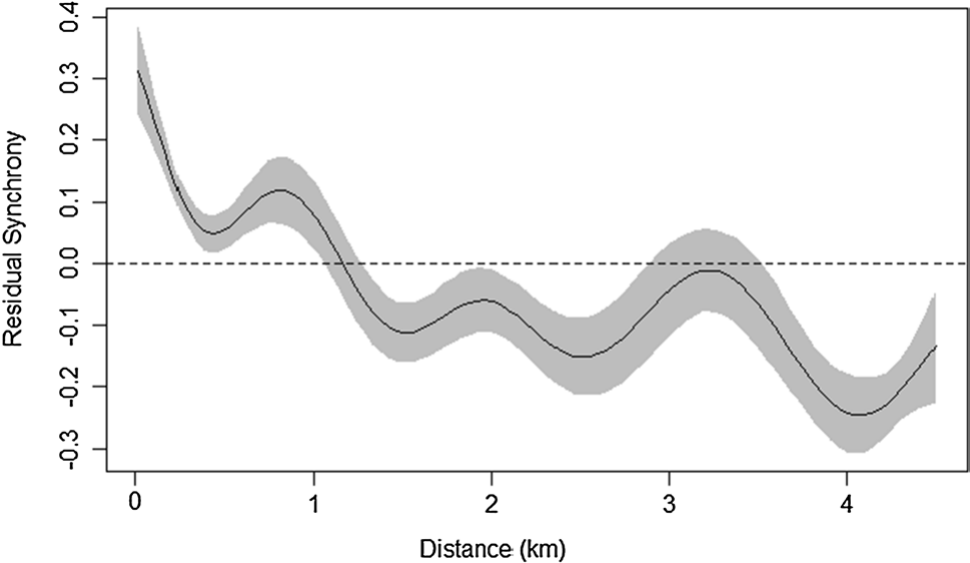
GAM showing residual population synchrony and distance between transect section pairs less than 4.5 km apart. Above the dashed line, population dynamics are synchronised; below the line, dynamics are asynchronous. The shaded area shows the 95% CI

**Fig. 4:**
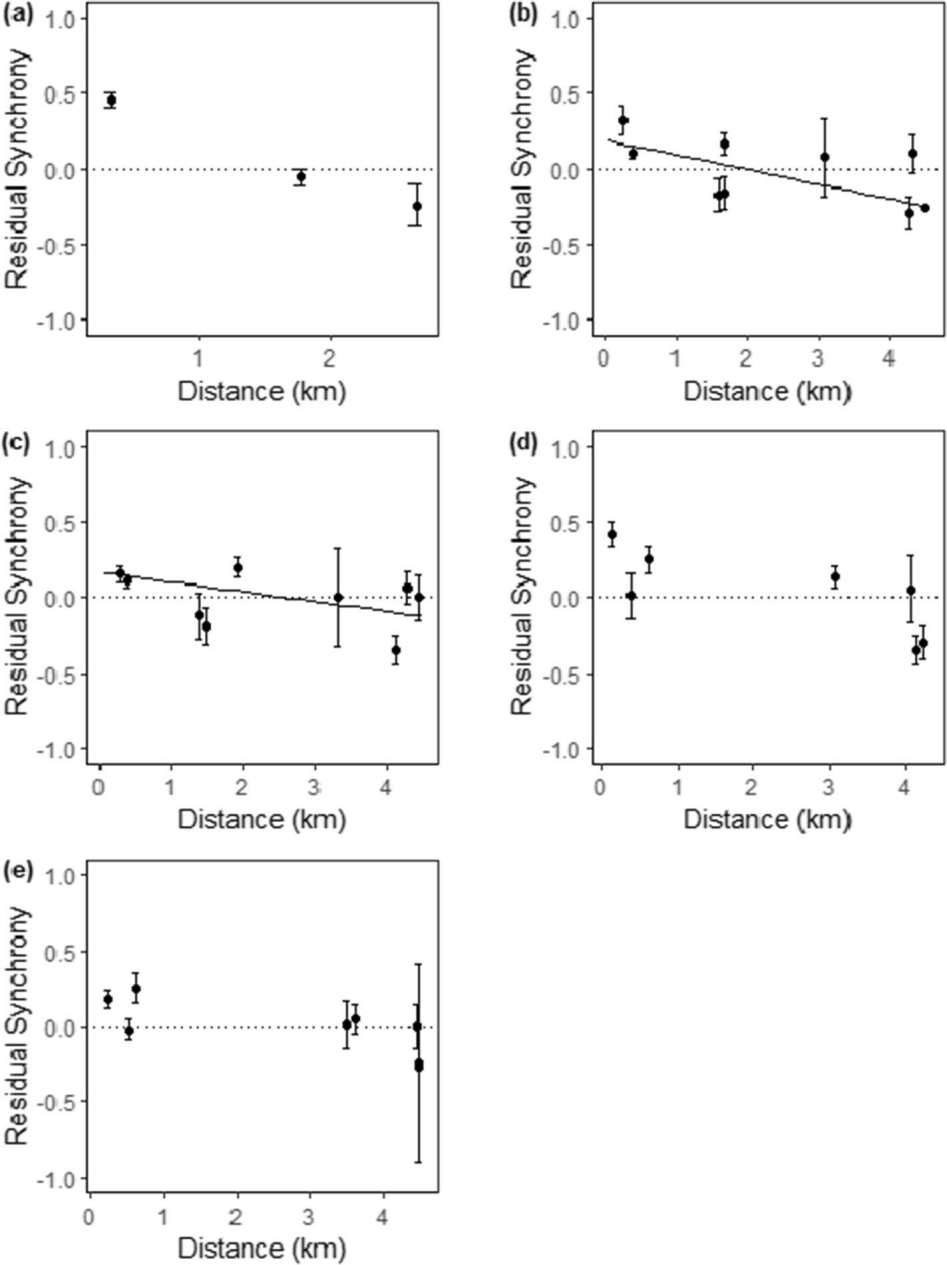
The relationship between residual population synchrony and distance for site specific comparisons between transect sections at **a** Site 2, **b** Site 8, **c** Site 9, **d** Site 14, **e** Site 15 and corresponding transect sections up to 4.5 km apart (both within the site and with sections from other sites). Points show average synchrony for each site pair. Error bars show the 95% CI. Above the dashed line, population dynamics are synchronised; below the line, dynamics are asynchronous. Site numbers as per Fig. 1

Butterfly transects are sub-divided into sections, so population synchrony was also assessed at this level. Synchrony between transect sections less than 4.5 km apart showed a significant trend with distance (GAM = edf: 8.662; Ref.df: 8.967; *F*: 26.85; *p* > 0.001). The trend was generally negative, though some fluctuations in synchrony were present (Fig. 3). Population synchrony showed a rapid decline with distance between 0 and 425 m, with synchrony falling close to 0 at around 425 m. There was an increase in synchrony between approximately 425–900 m, before a further decline. Populations showed fluctuating, asynchronous dynamics at distances above 1200 m, which could be due to site-specific factors. To investigate this further, models were built for key sites (those which had over 350 data points). Although comparisons between transect sections were only statistically significant for two of the five key sites analysed (Table 2), there was a general decline in synchrony with distance, with synchrony dropping to near 0 (representing uncorrelated dynamics) at around 2 km (Fig. 4). There was also some variation in the degree of synchrony between sites at similar distances, with population dynamics at some sites becoming asynchronous (negatively correlated), while others remained synchronised (Fig. 4).

**Table 2:** Distance effects on population synchrony (between transect section pairs) with separate models built for each selected site to incorporate all pairwise comparisons between transect sections less than 4.5 km apart (both within the selected site and with sections from other sites)

Predictor	Coefficient	SE	*t*	df	*p*
a) Site 2					
Distance (km)	− 0.083	0.044	− 1.880	163.324	0.062
b) Site 8					
Distance (km)	− 0.102	0.037	− 2.763	13.409	**0.016**
c) Site 9					
Distance (km)	− 0.100	0.033	− 3.057	16.127	**0.007**
d) Site 14					
Distance (km)	− 0.072	0.044	− 1.640	4.583	0.167
e) Site 15					
Distance (km)	− 0.035	0.026	− 1.352	7.508	0.216

Significant relationships are highlighted in bold

### Local-scale (within-site) synchrony

**Fig. 5:**
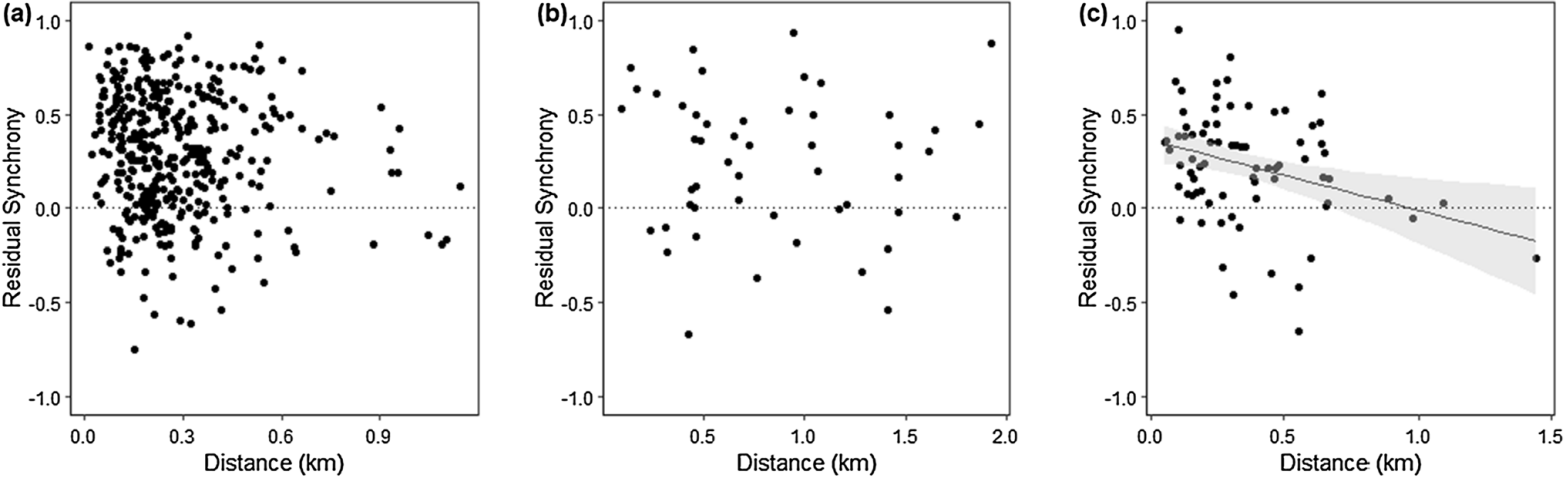
The relationship between residual population synchrony and distance for within-site transect section pairs with **a** closed habitat structure, **b** open, and **c** mixed habitat structure (closed vs open). The shaded area shows 95% CI. Above the dashed line, population dynamics are synchronised; below the line, dynamics are asynchronous

To investigate local-scale movement, population synchrony was assessed within sites (pairwise comparisons of sections within the same transect). To investigate the effect of habitat structure on population synchrony, the data were split based on the habitat of the pair of transect sections compared (open habitat, closed habitat, or mixed habitat) and analysed separately. For all transects, synchrony declined with distance between transect section pairs (Table 3; Online Resource 3). However, the relationship between distance and synchrony was dependent on the habitat structure of the pair of sections compared. Synchrony declined significantly with distance for sections with a mixed habitat structure but not for section pairs with similar habitat structure (Table 3; Fig. 5).

**Table 3:** Significance of habitat similarity and distance on local population synchrony (between pairs of sections within the same transect site) for (a) all section pairs within a transect site, (b) section pairs with a closed habitat structure, (c) section pairs with an open habitat structure, and (d) section pairs which had different habitat structures

Predictor	Coefficient	SE	*T*	df	*p*
a) All sections					
Distance (km)	− 0.117	0.059	− 1.956	352.314	**0.047**
b) Closed habitat sections					
Distance (km)	− 0.152	0.092	− 1.662	352.648	0.097
c) Open habitat sections					
Distance (km)	0.019	0.111	− 0.171	37.701	0.865
d) Mixed habitat sections					
Distance (km)	− 0.449	0.144	− 3.116	70.441	**0.003**

Significant relationships are highlighted in bold

## Discussion

Site-level analysis demonstrated that at large spatial scales (between sites), synchrony was not related to distance, while habitat similarity synchronised population dynamics, with sites sharing the same broad habitat type showing greater synchrony. By contrast, in populations located less than 4.5 km apart, synchrony marginally declined with distance, but there was no effect of habitat similarity. When studied at the transect-section level, trends in population synchrony related well with typical dispersal distance in the study species; significant declines in synchrony were present, with the most rapid declines within 1 km. At larger scales, comparisons of key sites revealed that synchrony over longer distances is site specific. Within-site assessments of synchrony highlighted differences in dispersal based on habitat structure, with declines in population synchrony only significant when comparing transect sections that had different habitat structures (closed vs open). Contrary to predictions, population synchrony was not significantly related to extinction risk.

In the current study, dispersal was shown to synchronise population dynamics at small scales (between transect sections), but not over larger distances (between sites). In the current study, the average distance between sites was 6.6 km. Although dispersal has been reported to synchronise dynamics over large spatial scales in mobile butterfly species, given that *B. euphrosyne* has a maximum dispersal distance of 4.5 km, it is perhaps not surprising that synchrony was not significantly related to distance between sites (Barnett and Warren 1995; Powney et al. 2011). Previous studies have also found synchrony to be dependent on spatial scale, with a greater rate of decline in synchrony at the local scale than between sites (Sutcliffe et al. 1996). While there is potential for habitat-driven asynchrony to be averaged out when studied at larger spatial scales, such as between transect sites rather than sections, mobility is also a key determinant on the rate of distance decay in synchrony. As the decline in synchrony with distance, at the transect section-level, relates well with reported movement in *B. euphrosyne,* it is likely attributable to a decline in movement frequency with distance, which would facilitate the decoupling of population dynamics (Barnett and Warren 1995). The rapid decline in synchrony with distance up to 425 m conforms well to the average movement distance of 315 m observed in a mark–release–recapture study of *B. euphrosyne* and suggests that dispersal is most common over short distances, such as occurs within-sites (Davidson 2017). Indeed, the average distance between transect sections within the same site in our study system is around 347 m, which supports this interpretation. Our results provide good evidence that residual population synchrony is an effective indicator of local-scale movement in sedentary species. However, an increase in synchrony between approximately 425 to 750 m was also observed; at these distances, most pairwise comparisons (64%) were of transect sections between- rather than within-site. Between sites, habitat management is likely to be independent, whereas rotational management within-sites can drive asynchrony if, for example, adjacent transect sections are at different stages of ecological succession following management. The slight increase in synchrony at distances where the transition is made from within- to between-site comparisons could be explained by habitat effects, with habitat-driven asynchrony being a less strong driver of population dynamics between sites than within.

The GAM also highlighted fluctuating, asynchronous dynamics above distances of approximately 1200 m. As dispersal between populations over these larger distances is likely to be very limited, these fluctuations in synchrony are best explained by variation in habitat type and management regime, rather than dispersal. The site-specific models supported this interpretation, with variation in the degree of synchrony between sites at similar distances. Although this species is reported to move as far as 4.5 km, these movements are likely rare, and habitat effects may offset any synchronising effects that dispersal would have on population dynamics at this scale. As a specialist of early successional habitat, *B. euphrosyne* populations are strongly influenced by vegetation successional stage. Abundance in woodland colonies is reported to peak around 2 years following coppice management, as succession progresses, breeding success declines as the habitat becomes unsuitable (Thomas et al. 1991; Barnett and Warren 1995). Therefore, where sites are managed over different timescales (for example, an uncoordinated programme of scrub clearance or coppicing), variation in successional stage may drive asynchrony between populations occupying different management units. This has been observed in the other early successional butterfly species such as the heath fritillary, *Melitaea athalia*, as well as *P. argus* where neighbouring colonies occupying patches of heathland at different successional stages were shown to fluctuate asynchronously (Warren 1987; Thomas et al. 1991).

Habitat similarity was shown to have a synchronising effect on *B. euphrosyne* populations over large spatial scales, this result is consistent with the previous research in other butterfly species (Powney et al. 2010). In our study region, in addition to breeding in early successional woodland and bracken habitats, such as in the south of England, *B. euphrosyne* also occupies well-drained grassland, scree, and scrub habitats (Barnett and Warren 1995). Limestone grasslands in this region are reported to regenerate slowly and thus may provide suitable breeding habitat for an extended period (Barnett and Warren 1995). Slow regeneration of habitat may be associated with greater population stability, which is likely to promote synchrony in population dynamics (Sutcliffe et al. 1996). Habitat-dependent carrying capacities or responses to environmental perturbations are also potential mechanisms that could drive stronger synchrony in populations occupying sites with the same habitat type (Powney et al. 2010, 2011). Although similarity in broad habitat type between transect sites was demonstrated to have a synchronising effect on population dynamics at large spatial scales, no significant effect of habitat similarity was found when comparing sites less than 4.5 km apart. Again, at these smaller distances, differences in timing of habitat management, which are likely to generate heterogeneity in the successional stage of vegetation, may have decoupled population dynamics between sites sharing the same broad habitat type. Conversely, when synchrony is examined at larger scales, the timing of management is likely to be independent, and therefore, management-driven heterogeneity may be averaged out (Sutcliffe et al. 1996).

Population synchrony was also used to investigate variation in *B. euphrosyne* dispersal, based on habitat structure. Habitat structure was predicted to influence synchrony, with stronger distance decay predicted in closed habitats (e.g., woodland), than in those with a more open structure (e.g., grassland). This prediction was based on previous research on other butterfly species, showing that populations occupying grassland-dominated transects were characterised by more synchronised dynamics than those in woodland transects (Powney et al. 2012). Our prediction was also based on the current understanding of the mobility of the species. *B. euphrosyne* is described as highly sedentary in woodland habitats, where it forms discrete colonies with limited dispersal (Barnett and Warren 1995). By contrast, in open habitats, where there are fewer barriers to dispersal, the butterfly is thought to be more mobile and to form metapopulations. Indeed, the results here show population synchrony in open habitats to be independent of distance, while synchrony showed a tendency to decline with distance in closed habitats. In open habitats, individuals may be able to move freely between transect sections over the distances studied, which would synchronise population dynamics equally. Whereas, woodland habitats are likely to pose greater barriers to dispersal. The rapid decline in synchrony with distance observed between transect section pairs with contrasting habitat structures (mixed) may indicate that individuals are unwilling to move between habitats. Studying functional measures of distance and the permeability of matrix habitat, in addition to habitat patch characteristics and Euclidean distance, as used here, may allow for better understanding of the landscape features that promote or inhibit dispersal. However, our results suggest that *B. euphrosyne* populations occupying different habitats are likely to be effectively isolated, as variation in habitat structure poses a barrier to dispersal, even over relatively short distances.

No relationship between average site synchrony and population status was found. Although we predicted that population persistence would be associated with higher average site synchrony, asynchrony also is important for persistence at the metapopulation level, as it reduces the likelihood of simultaneous extinction and can facilitate recolonisation (Hanski 1998). Many of the *B. euphrosyne* populations studied are small (over the study period 60% of annual site indices were ≤ 50). Asynchrony between populations may be particularly important for species with low abundance, as these are likely to be more vulnerable to extinctions associated with local stochasticity than those with higher abundance. Furthermore, landscape connectivity may facilitate the dispersal of parasites and natural enemies between populations. Potentially, any beneficial effects of dispersal on population extinction risk could be offset by these factors. Pre-whitening of the data allowed the synchronising effects of shared climate to be controlled for, but the potential roles of biotic interactions, such as the movement of natural enemies between populations, are more difficult to account for (Oliver et al. 2017). Although synchrony was not found to relate to extinction risk here, given the role of habitat in synchronising population dynamics, landscape changes such as biotic homogenisation may increase population synchrony and therefore future vulnerability to extinction (Powney et al. 2010; Pandit et al. 2016). Studying temporal change in population synchrony, particularly following habitat fragmentation or the extinction of one or more patches in a network, could further our understanding of the impacts of environmental change on dispersal and population persistence. The sensitivity of population synchrony to local-scale and likely, infrequent, dispersal in the current study shows that synchrony has utility for assessing functional connectivity in sedentary species. While population synchrony is interpreted as the result of local dispersal in *B. euphrosyne*, beyond management units, population dynamics are likely to be influenced by vegetation successional stage, with asynchrony between units driven by timing of the management regime. Although this study shows no clear implications of synchrony on population persistence at the site level, these results have important implications for landscape design and management. Asynchrony is key for regional metapopulation persistence and habitat management may be used as a tool to maintain asynchrony between populations, decreasing the risk of simultaneous extinction. The strong distance decay in synchrony between transect sections with contrasting habitat structures also observed suggests that, even over short distances, populations occupying patches with different habitat types can be functionally isolated. These results highlight the need to consider habitat structure during management planning so as to ensure that rotational management produces habitat that is readily colonisable.

## Supplementary Information

Below is the link to the electronic supplementary material.

Supplementary file1 (DOCX 42 KB)

## Data Availability

Transect section-level abundance counts were requested directly from the United Kingdom Butterfly Monitoring Scheme. Site abundance indices and national collated indices are available from: https://doi.org/10.5285/5c9c946d-34f8-4afb-83e3-f0cbc7123fec and https://doi.org/10.5285/ace3c3ef-df89-40b9-ba8b-106997fd6d9c

## Code availability

R code used during the current study is available from the corresponding author on reasonable request.
