# Supplementary material for: Population synchrony indicates functional connectivity in a threatened sedentary butterfly
Source: Oecologia. 2023 Mar 28;201(4):979–89. doi: 10.1007/s00442-023-05357-2 (PMC10113297; doi:10.1007/s00442-023-05357-2)
Supplement: Supplementary file 1 — Supplementary file1 (DOCX 42 KB) [file 442_2023_5357_MOESM1_ESM.docx]

**Supporting Information**

Table S1. Site details of the 15 UKBMS butterfly transects used in synchrony analyses. Classifications of habitat type were made based on the dominant and secondary habitats present at the site level using the 2015 Land Cover Map (Rowland et al. 2017). Site 10 (Leighton Moss) is predominantly reed bed and as *B. euphrosyne* was only regularly recorded on two of the transect sections, habitat type refers to these sections only.

| **Site Number** | **Transect Name** | **Grid Reference** | **Number of Sections** | **Transect Length (m)** | **Dominant Habitat Type** | **Secondary Habitat Type** |
| --- | --- | --- | --- | --- | --- | --- |
| 1 | Whitbarrow - Howe Ridding NNR | SD434881 | 15 | 1410 | Broadleaved Woodland | NA |
| 2 | Whitbarrow North | SD4488 | 15 | 2870 | Broadleaved Woodland | Acid Grassland |
| 3 | Whitbarrow NNR - Hervey CWT | SD441869 | 11 | 4000 | Acid Grassland | Broadleaved Woodland |
| 4 | Whitbarrow NNR - Farrer's Allotment | SD452855 | 15 | 4100 | Acid Grassland | Broadleaved Woodland |
| 5 | Heathwaite NT | SD448768 | 7 | 3500 | Broadleaved Woodland | Calcareous Grassland |
| 6 | Arnside Knott NT | SD453773 | 12 | 4700 | Broadleaved Woodland | NA |
| 7 | Eaves Wood NT | SD466762 | 11 | 4500 | Broadleaved Woodland | NA |
| 8 | Gait Barrows | SD477771 | 9 | 1400 | Broadleaved Woodland | NA |
| 9 | Gait Barrows NNR (Warden's) | SD481773 | 15 | 1900 | Broadleaved Woodland | Arable |
| 10 | Leighton Moss | SD487760 | 15 | 5922 | Broadleaved Woodland | Improved Grassland |
| 11 | Yealand Hall Allotment | SD489762 | 14 | 4000 | Broadleaved Woodland | NA |
| 12 | Holme Park Fell | SD542794 | 10 | 3315 | Calcareous Grassland | Calcareous Grassland |
| 13 | Warton Crag RSPB | SD489727 | 7 | 2800 | Broadleaved Woodland | NA |
| 14 | Warton Crag LWT | SD493730 | 15 | 5400 | Broadleaved Woodland | NA |
| 15 | Warton Crag LNR | SD494726 | 15 | 3181 | Broadleaved Woodland | Calcareous Grassland |


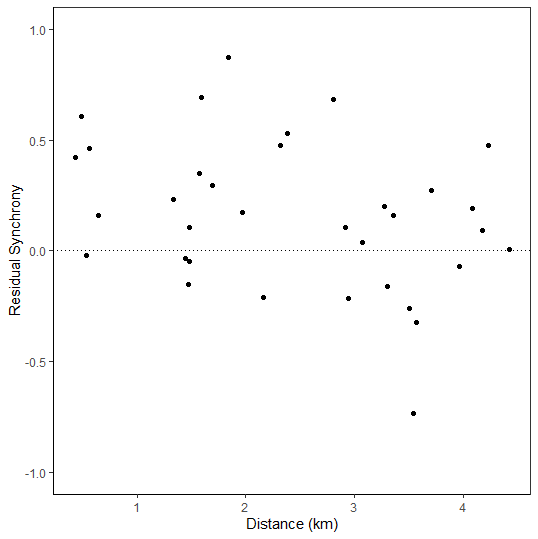


Figure S1: Residual population synchrony and distance. Site-level pairwise comparisons were made between 15 UKBMS transects, for site pairs less than 4.5 km apart. The shaded area shows the 95% CI. Above the dashed line population dynamics are synchronised, below the line dynamics are asynchronous.


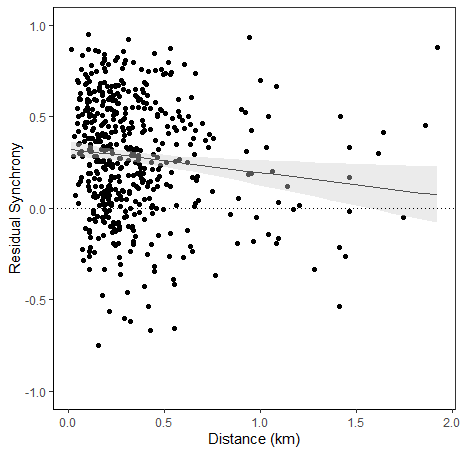


Figure S2. The relationship between residual population synchrony and distance for all within-site comparisons. Above the dashed line population dynamics are synchronised, below the line dynamics are asynchronous. The shaded area shows 95% CI.
